# Validation of the MUSIC Model of Academic Motivation Inventory: Evidence for Use With Veterinary Medicine Students

**DOI:** 10.3389/fvets.2019.00011

**Published:** 2019-02-06

**Authors:** Brett D. Jones, Meghan K. Byrnes, Mia W. Jones

**Affiliations:** ^1^School of Education, Virginia Tech, Blacksburg, VA, United States; ^2^Blacksburg High School, Blacksburg, VA, United States

**Keywords:** MUSIC model of motivation, motivation, engagement, assessment, motivating students, student perceptions, MUSIC Inventory

## Abstract

The MUSIC® Model of Motivation is used to help instructors select strategies that can increase students' motivation and engagement in courses. The MUSIC model is comprised of five categories of strategies titled: empowerment, usefulness, success, interest, and caring. The purpose of this study was to examine the extent to which the MUSIC® Model of Academic Motivation Inventory (College Student version, short-form), demonstrates acceptable psychometric properties when used with students enrolled in a College of Veterinary Medicine. The inventory is comprised of five scales that correspond to the five MUSIC model components, and it measures the extent to which students perceive that: they have control in the course (empowerment); the activities in the course are useful to their future (usefulness); they can succeed in the course (success); the teaching activities and coursework are interesting (interest); and the instructor cares about students' learning and well-being (caring). The inventory has been validated for use with many different student populations, including students in different countries and of different ages (e.g., college students, middle and high school students, elementary school students). However, the inventory has not been validated for use with veterinary medicine students. We analyzed the data from 578 questionnaires that were obtained from students in six different courses at a College of Veterinary Medicine. We examined the psychometric properties of the MUSIC inventory by: (a) computing the internal consistency reliabilities for the scales; (b) calculating the fit indices and factor loadings obtained from confirmatory factor analyses; and (c) computing correlation coefficients between the inventory scales and students' self-reported effort in the course. The results provide evidence that the inventory demonstrates acceptable psychometric properties for use with veterinary medicine students. Consequently, the MUSIC Inventory can be used by researchers and instructors to assess students' motivation-related perceptions of courses.

## Introduction

The MUSIC® Model of Motivation [MUSIC model; (1, 2)] is an organizational framework that can be used by instructors and researchers to design motivating instruction and to examine students' motivation in educational settings. The basic principles of the MUSIC model are that

“the instructor needs to ensure that students: (1) feel *empowered* by having the ability to make decisions about some aspects of their learning, (2) understand why what they are learning is *useful* for their short- or long-term goals, (3) believe that they can *succeed* if they put forth the effort required, (4) are *interested* in the content and instructional activities, and (5) believe that others in the learning environment, such as the instructor and other students, *care* about their learning and about them as a person” [(2), p. 9, MUSIC is an acronym for these principles].

The MUSIC model was developed based on research and has been used in a variety of ways in higher education, such as (a) to investigate students' motivation-related perceptions in courses or programs (3–6), (b) to design or redesign courses (7, 8), (c) to develop motivational interventions to improve instruction (9), and (d) to investigate the relationships between students' course perceptions and their identification with a domain (e.g., their value for engineering) and career goals (10, 11).

For these uses of the MUSIC model, it is often necessary for instructors and researchers to *assess* students' MUSIC-related perceptions in a course (e.g., perceptions of the usefulness of the course). The MUSIC® Model of Academic Motivation Inventory [the “MUSIC Inventory” (12)] “measures the five primary components of the MUSIC Model of Motivation: empowerment, usefulness, success, interest, and caring” [(12), p. 4]. The College Student version of the MUSIC Inventory includes 26 items that comprise five scales (i.e., empowerment, usefulness, success, interest, and caring). Each item is rated using a 6-point Likert-format scale that ranges from 1 (*strongly disagree*) to 6 (*strongly agree*). A mean scale score is obtained by averaging the items in each scale.

Researchers have validated the College Student version of the MUSIC Inventory for use with undergraduate students in the U.S. (13, 14), China (15), Colombia (15), and Egypt (16). Less is known about the validity of the MUSIC Inventory when used for post-graduate students, such as those in professional schools. One study that examined the validity of the MUSIC Inventory for professional students found that it produced valid and reliable scores when used with student pharmacists (17). The authors of that study also concluded that it was practical for instructors to use and suggested that the MUSIC Inventory could provide useful information that instructors could use to redesign courses or to help struggling students.

## Purpose and Research Question

Given the potential benefits of using the MUSIC model and associated MUSIC Inventory with students in professional schools, we sought to determine the extent to which the MUSIC Inventory would produce valid and reliable scores when used with veterinary medicine students. Because the MUSIC Inventory has been successfully validated with samples of students with various levels of education, including those in professional schools [e.g., (17)], we hypothesized that it would also be valid for use with veterinary medicine students. Therefore, our research question was: To what extent does the MUSIC Model of Academic Motivation Inventory produce valid scores when used with veterinary medicine students?

Pace et al. (17) found that the 26-item College Student version of the MUSIC Inventory was easy to use and took little time to administer. However, in our experience as instructors and researchers (and as noted by Pace et al.), the MUSIC Inventory items are often incorporated into a questionnaire that includes other items that assess other aspects of students' course perceptions. For example, Jones (18) asked students to rate the instructor, course, and amount of effort they put into the course. Other instructors and researchers have also included open-ended items, such as “How will what you have learnt be useful during your studies and your professional life?” (6). Because students can experience survey fatigue when they are asked too many questions in one survey or are asked to complete multiple surveys (19), we wanted to examine the validity of the 20-item short-form of the MUSIC Inventory (12). Although the short-form is only six items shorter (23% shorter) than the 26-item version, if it is found to be valid, it could reduce survey fatigue or allow instructors and researchers to include other items on a questionnaire in place of those six items without increasing the length of the questionnaire. Therefore, we chose to investigate the validity of using the 20-item short-form of the MUSIC Inventory with veterinary medicine students.

## Materials and Methods

### Participants and Procedure

Participants were enrolled as first- or second-year students in the College of Veterinary Medicine at a large, public university in the Eastern United States. Students completed an online questionnaire near the end of their course about their perceptions of the course. At the end of the questionnaire, students were invited to give their consent to allow their questionnaire responses to be used as part of our study. Only students who gave consent to participate in the study were included. This study protocol was approved by the Institutional Review Board (IRB) at the institution of the lead authors.

We included the responses of students from six different courses and each course was comprised of about 132 students. The courses were typical of those offered by veterinary schools in the U.S. and included topics such as anatomy, physiology, radiology, histology, nutrition, pharmacology, immunology, communication skills, and professionalism.

The number of students in each course who completed all of the questionnaire items and provided consent to participate in the study ranged from 82 (62.1%) to 106 (80.3%) (see Table 1). More students were female (440 students = 76.1%) than male (134 students = 23.2%); two students (0.3%) reported “Other” for their sex, and two students (0.3%) did not report their sex. Students reported their race/ethnicities as follows: 450 (77.9%) White or Caucasian (not Hispanic), 39 (6.7%) Asian or Pacific Islander, 30 (5.2%) Hispanic, 28 (4.8%) more than one ethnicity, 23 (4.0%) Black or African American, 4 (0.7%) another race/ethnicity not provided as an option, 2 (0.3%) Native American, and 2 (0.3%) did not report their race/ethnicity. The average age was 25 years old for all students (participants and non-participants) who matriculated at this college.

**Table 1:** Information about the courses and participants' response rates.

**Course no**.	**Year of student**	**Semester**	**Course topic**	***n***	**% response**	**Study sample**
1	1	First fall	Foundations of medicine	106	80.3	1
2	2	First fall	Circulatory system	102	77.3	1
3	1	Second fall	Host and defense	82	62.1	2
4	2	Second fall	Gastrointestinal system	88	66.7	2
5	1	First spring	Non-technical competencies	95	72.0	3
6	2	First spring	Reproduction	105	79.5	3

### Instruments

#### MUSIC Model of Academic Motivation Inventory (College Student Short-Form Version)

The MUSIC Inventory (College Student short-form version) includes 20 items that are used to create five scales (i.e., empowerment, usefulness, success, interest, and caring), each comprised of four items (12). The response options for each item are as follows: *1* = *Strongly Disagree, 2* = *Disagree, 3* = *Somewhat Disagree, 4* = *Somewhat Agree, 5* = *Agree, 6* = *Strongly Agree*. A mean scale score is computed by averaging the items in each scale. For example, the four interest items are averaged to obtain a mean scale score for interest. The scales measure “the degree to which a student perceives that: he or she has control of his or her learning environment in the course [empowerment]; the coursework is useful to his or her future [usefulness]; he or she can succeed at the coursework [success]; the instructional methods and coursework are interesting [interest]; and the instructor cares about whether the student succeeds in the coursework and cares about the student's well-being [caring]” [(12), p. 5]. The complete MUSIC inventory is available online at Jones (12) and example items include the following: “I have flexibility in what I am allowed to do in this course” (empowerment), “In general, the coursework is useful to me” (usefulness), “I am confident that I can succeed in the coursework” (success), “The coursework is interesting to me” (interest), and “The instructors care about how well I do in this course” (caring). Researchers have found that the MUSIC Inventory is reliable and valid for use with many different types of students and courses (13, 14, 18).

#### Course Effort

To measure students' self-reported course effort, we used the 4-item Course Effort scale that assesses the amount of effort that students believe they are putting into a course (Jones, unpublished manuscript). The scale consists of four items rated on a six-point Likert-format scale: 1 = *Strongly Disagree*, 2 = *Disagree*, 3 = *Somewhat Disagree*, 4 = *Somewhat Agree*, 5 = *Agree*, 6 = *Strongly Agree*. An example item is, “In this course, I put forth my maximum effort.” Prior studies have documented acceptable reliability estimates (e.g., Jones, unpublished manuscript; α = 0.93) and the values in the present study were also very good to excellent (α = 0.87 for sample 1; α = 0.90 for sample 2; and α = 0.90 for sample 3).

In addition to the quantitative Course Effort scale items, we included an open-ended item that asked students to explain *why* they put forth the amount of effort that they put into the course. We selected two of the six participating courses through purposive sampling based on perceived course difficulty and content: (a) one course was considered “easier” by students and the course content was related to students' non-technical competencies (such as professionalism and ethics, as opposed to content directly related to treating animals), and (b) another course was perceived to be more challenging to students and the content directly related to treating animals.

#### Course Rating

Students' overall rating of the course was assessed with an item that has been used in other studies [e.g., (18)] and that is similar to questions that are used as part of end-of-course evaluations at some colleges. The item and rating scale are as follows: “My overall rating of the course” (*1* = *Terrible, 2* = *Very poor, 3* = *Poor, 4* = *Good, 5* = *Very good, 6* = *Excellent*).

### Analysis and Interpretation of Values

We assessed the internal consistency reliability for all of the MUSIC Inventory scales by calculating Cronbach's alpha values using SPSS (version 23). We used the criteria provided by Kline (20) to assess the alpha values in our study: >0.9 was considered excellent, between 0.7 and 0.9 was good, between 0.6 and 0.7 was acceptable, and <0.6 was deemed to be unacceptable.

To examine how the MUSIC Inventory items in our study fit the five-factor structure of the MUSIC model, we conducted confirmatory factor analyses (CFAs) using LISREL (version 8.80). We used three fit indices to assess the results of the CFA: the Comparative Fit Index (CFI), the Standardized Root Mean Square Residual (SRMR), and the Root Mean Square Error of Approximation (RMSEA). Based on Hu and Bentler (21), CFI values closer to 1 indicate a better fit, with values above 0.90 representing a reasonable fit and above 0.95 representing a good fit. For the SRMR, values closer to 0 indicate a better fit, with values <0.08 indicating a good fit (21). Similar to the SRMR, values closer to 0 indicate a better fit for the RMSEA, with values <0.08 indicating a reasonable fit and values <0.05 indicating a good fit (20, 22, 23).

We computed Pearson's correlation coefficients for the MUSIC scales and, based on prior studies (13), we expected the scales to be moderately correlated. Next, we examined the factor loadings to determine the extent to which each item loaded on the factor we expected. For example, the caring items should load appropriately on the caring factor. We determined that the factor loadings were acceptable when they were >0.32 (24).

To provide evidence for the predictive validity of the scales, we computed Pearson's correlation coefficients using SPSS (version 23) to assess the relationship between the MUSIC Inventory scales and students' course effort. Because increases in students' MUSIC perceptions should lead to greater effort (1, 2, 13), we predicted that the MUSIC Inventory components would correlate positively with students' self-reported course effort and overall course rating (18). We used the following criteria, based on Cohen (25), to assess the significance of the correlations: a large effect size is 0.50 or greater, a medium effect size is 0.30–0.49, and a small effect size is 0.10–0.29.

The analysis of the open-ended item (that asked students why they put forth the amount of effort they put forth in the course) involved an analysis of the students' responses based on a grounded theory approach (26, 27). Two of us authors developed the initial coding scheme after reading all of the responses from one course at the veterinary college that was not included in the present study. We identified themes in the responses and created coding categories within each of the themes. Next, we independently coded all of the responses in two of the courses included in this study. Coding disagreements were settled by mutual consent. We developed 16 coding categories that were grouped into two themes: responses related to why students put forth effort and responses related to why students did not put forth effort. We computed the inter-rater reliability rate as a percentage using this formula: 100 – 100^*^(number of coding disagreements between coders/number of times that the codes were utilized). The inter-rater reliability rate was 89.5% for Course 5 and 90.8% for Course 6 (see Table 1 for information about Course 5 and 6).

To obtain sufficient statistical power and precision to conduct the CFAs (which were the analyses that required the largest number of participants), we sought a sample size of about 200. Although no single criterion can be used to determine a sufficient sample size for a CFA, common rules of thumb used by researchers include: (a) obtaining a minimum of 100 to 200 cases (28–30) or (b) obtaining a subject-to-variable ratio of not less than 5:1 or 10:1 (31, 32), which would be equivalent to 100 to 200 participants for the 20-item MUSIC Inventory used in the present study. Because the number of students in each course was at the low end or below these ranges (*n* ranged from 82 to 106; see Table 1), we combined two courses to obtain sample sizes closer to 200 (i.e., 208, 170, and 200). We combined one course of first-year students with one course of second-year students to avoid a sample that included the same individual twice. Using this method, we created three samples (see Table 1) and conducted the analyses described in this section for each of the three samples.

## Results

We used the Cronbach's alpha values shown in Table 2 to assess the reliability of each of the MUSIC Inventory scales. The alpha values ranged from 0.78 to 0.93; and therefore, we deemed the reliability of the scales to be good to excellent (20). To compare these results to those obtained in other studies, Table 2 also includes the alpha values that were reported for the 26-item College Student version of the MUSIC Inventory, which was used with undergraduate students [(13), p. 5] and student pharmacists [(17), p. 595].

**Table 2:** Cronbach's alpha values and fit indices.

		**Cronbach's alpha values**							
**Sample**	***n***	**M**	**U**	**S**	**I**	**C**	**CFI**	**SRMR**	**RMSEA**
1	208	0.82	0.85	0.88	0.78	0.81	0.98	0.057	0.054
2	170	0.85	0.88	0.88	0.80	0.86	0.96	0.069	0.080
3	200	0.90	0.93	0.92	0.87	0.84	0.97	0.065	0.073
Jones^a^	338	0.91	0.96	0.93	0.95	0.93	0.92	0.055	0.085
Pace^a^	154	0.89	0.91	0.92	0.91	0.92	–	–	–

*CFI, RMSEA, and SRMR are values from CFAs that were conducted with the 20-item College Student short-form of the MUSIC Inventory comprised of five scales (i.e., empowerment [M], usefulness [U], success [S], interest [I], and caring [C])*.

a *Values for the “Jones” and “Pace” sample were reported in (13) and (17), respectively, for the 26-item College Student version of the MUSIC Inventory and are not based on data collected in the present study*.

The MUSIC Inventory scales were moderately correlated with each other as expected, with most correlations falling in the range of 0.30 to 0.60 (see Table 3). The fit indices from the CFAs are shown in Table 2, in addition to the values from the 26-item MUSIC Inventory reported in Jones and Skaggs [(13), p. 5]. For the short-form examined in the present study, the CFI values indicate a good fit (21), the SRMR values indicate a good fit (21), and the RMSEA values indicate a reasonable fit (22). The CFA factor loadings (see Table 4) ranged from 0.63 to 0.91, which demonstrates that the items loaded very well on the appropriate factors (24).

**Table 3:** Pearson's correlation coefficients for the study variables.

	**Empowerment**	**Usefulness**	**Success**	**Interest**	**Caring**	**Effort**
Usefulness	0.21, 0.14, **0.35**	–	–	–	–	–
Success	**0.43, 0.38, 0.42**	**0.30, 0.40**, 0.11	–	–	–	–
Interest	**0.40, 0.39, 0.48**	**0.56+, 0.56+, 0.79+**	**0.50+, 0.56+**, 0.13	–	–	–
Caring	**0.40, 0.30, 0.53+**	**0.36, 0.49, 0.38**	**0.40, 0.51+, 0.46**	**0.47, 0.53+, 0.49**	–	–
Effort	**—**, **—**, **—**	0.23, 0.24, **0.44**	−0.10, 0.12, −0.20	0.15, 0.26, **0.45**	**—**, 0.22, 0.18	–
Rating	0.28, 0.13, **0.43**	**0.30, 0.34, 0.60+**	**0.40, 0.48**, 0.20	**0.49, 0.43, 0.68+**	**0.41, 0.35, 0.44**	**—**, **—**, **0.30**

*The three numbers in each cell are the correlation coefficients from three different analyses: sample 1 (n = 208), sample 2 (n = 170), and sample 3 (n = 200). Cohen's (25) criteria is indicated as follows: bold numbers with the “+” indicate a large effect size (0.50 or greater), bold numbers without the “+” indicate a medium effect size (0.30 to 0.49), and numbers not in bold indicate a small effect size (0.10 to 0.29). Statistically insignificant values are not included in the table and are represented as “**—**”*.

**Table 4:** Standardized factor loadings from the CFAs.

**Item**	**Empowerment**	**Usefulness**	**Success**	**Interest**	**Caring**
M1	0.74,0.81,0.83	–	–	–	–
M2	0.75,0.78,0.84	–	–	–	–
M3	0.67,0.71,0.83	–	–	–	–
M4	0.76,0.76,0.82	–	–	–	–
U1	–	0.74,0.66,0.89	–	–	–
U2	–	0.65,0.84,0.90	–	–	–
U3	–	0.86,0.86,0.85	–	–	–
U4	–	0.83,0.85,0.86	–	–	–
S1	–	–	0.74, 0.66, 0.89	–	–
S2	–	–	0.65, 0.84, 0.90	–	–
S3	–	–	0.86, 0.86, 0.85	–	–
S4	–	–	0.83, 0.85, 0.86	–	–
I1	–	–	–	0.63,0.79,0.76	–
I2	–	–	–	0.65,0.73,0.76	–
I3	–	–	–	0.73,0.69,0.83	–
I4	–	–	–	0.70,0.63,0.82	–
C1	–	–	–	–	0.67,0.78,0.77
C2	–	–	–	–	0.71,0.71,0.75
C3	–	–	–	–	0.74,0.80,0.78
C4	–	–	–	–	0.79,0.82,0.79

*The three numbers in each cell are the standardized coefficients from three different analyses: sample 1, sample 2, and sample 3*.

To examine the predictive validity of the MUSIC Inventory components, we correlated the MUSIC scales with students' self-reported course effort and course rating. The correlation coefficients between the MUSIC scales and course effort were fairly low and ranged from −0.20 to 0.45 (see Table 3). The correlation coefficients between the MUSIC scales and course rating ranged from 0.13 to 0.68, which indicated a moderate correlation (see Table 3).

Table 5 shows the results of the open-ended item that asked students why they spent the amount of time that they spent on the course. The codes are separated into two categories: (1) why students put forth effort and (2) why students did *not* put forth effort. Each student response was coded with at least one code and some responses were coded with two or three codes. No one response was coded with the same code twice. About a fifth of the students put forth effort because they wanted to perform well or achieve good grades or a high-class rank. About a third of the students in Course 5 reported that they did not need to put forth much effort because the course was easy.

**Table 5:** Percentage and number of responses for each code.

**Codes**	**% (No.)**	
	**Course 5**	**Course 6**
**WHY STUDENTS PUT FORTH EFFORT**		
Want to perform well, or achieve good grades or high-class rank	21.8% (27)	17.0% (24)
Want to learn the material, information important for their future, or value their education	8.9% (11)	24.1% (34)
Not applicable or didn't answer the question	4.8% (6)	4.3% (6)
Want to pass class, or afraid of failing	4.0% (5)	2.1% (3)
Material is difficult, or lots of work or information	0% (0)	14.2% (20)
The course or material is interesting or enjoyable	0% (0)	8.5% (12)
Slow at studying	0.8% (1)	1.4% (2)
**WHY STUDENTS DID NOT PUT FORTH EFFORT**		
Course was easy, or not much time needed to study	32.3% (40)	2.1% (3)
Had to focus on other classes	14.5% (18)	1.4% (2)
Want time for activities outside of class or want work-life balance	4.0% (5)	14.2% (20)
Want a healthy lifestyle physically and/or mentally (want less stress)	0% (0)	5.7% (8)
Tired of studying or school (burned-out)	0% (0)	2.1% (3)
Not useful to study (pointless)	5.6% (7)	1.4% (2)
Topics not interesting	3.2% (4)	1.4% (2)
Total	100% (124)	100% (141)

## Discussion

The purpose of this study was to examine the extent to which the MUSIC Model of Academic Motivation Inventory (College Student version, short-form) demonstrates acceptable psychometric properties when used with students enrolled in a College of Veterinary Medicine. To accomplish this purpose, we conducted the following analyses: (a) we calculated Cronbach's alpha values for each MUSIC Inventory scale to assess the reliability of the scales, (b) we computed the fit indices and factor loadings using CFA to assess the construct validity of the scales, and (c) we calculated correlation coefficients between the MUSIC Inventory scales and measures of effort and course quality to assess the predictive validity of the scales. In this section, we discuss the results of each of these three analyses and how they can be interpreted to provide evidence for the validity of the MUSIC Inventory.

### Reliability of the MUSIC Inventory

All of the Cronbach's alpha values were found to be acceptable, which indicates that the four items within each scale are highly correlated as expected. The Cronbach's alpha values were also fairly consistent across all three samples (see Table 2), as is evidenced by the fact that the range of values within any one scale across samples 1, 2, and 3 varied <0.09 (e.g., the range for empowerment was 0.08 because the lowest value was 0.82 for Sample 1 and the highest value was 0.90 for Sample 3).

Table 2 also includes Cronbach's alpha values obtained in two other studies that used the 26-item College Student version of the MUSIC Inventory: Jones and Skaggs (13) and Pace et al. (17). Almost all of the alpha values in these two studies were higher than the values obtained in the present study (see Table 2). This finding may be explained, in part, by the fact that the 26-item version used in the Jones and Skaggs study and the Pace et al. study includes more items for all of the scales except the success scale. Alpha values for scales tend to increase as the number of items in the scale increases; therefore, it is possible that the increase in alpha values is partly due to the larger number of items in each scale. Consistent with this explanation is the fact that the alpha values for the success scale—which included 4 items in both the short and full versions—were more similar for the 20-item and 26-item versions than they were for the empowerment, usefulness, interest, and caring scales. However, it is also possible that the alpha values are lower in the short-form for reasons other than the fact that there are fewer items in four of the scales in the present study. Regardless of why the alpha values for the short-form are lower, the values obtained in the present study are “good,” which indicates that the MUSIC Inventory scales produce reliable scores.

### Construct Validity of the MUSIC Inventory

The MUSIC scales were moderately correlated with each other (see Table 3), with only one of the correlations across all three samples > 0.56 (the correlation between usefulness and interest was 0.79 for Sample 3). Looked at another way, a correlation of 0.56 indicates that 31% of the variance is shared among variables (0.56^2^ = 31%), which indicates that over two-thirds (69%) of the variance was *not* shared. We expected the scales to be somewhat correlated because these constructs have been shown to be related (18). For example, one study found that telling students about the usefulness of a task increased their interest in the task, but only when they also had higher perceptions of success related to the task (33). If the correlations between the MUSIC scales in the present study had been very high, however, it would have indicated that the scales did not measure different constructs and would suggest that the highly correlated constructs could be combined into one scale. Thus, the moderate correlations obtained in the present study provide evidence for divergent validity because they indicate that the scales represent related, but different, constructs.

We conducted one CFA for each sample of students to examine how the items in the MUSIC Inventory fit the five-factor structure of the MUSIC model. We assessed the fit using three different fit indices and the item factor loadings because no one specific test provides enough information to adequately assess the data fit to the model. Data from all three samples fit the five-factor structure of the MUSIC model well (see Tables 2, 4): the values for the CFI for all three samples were >0.95, which indicates a good fit (21); the values for the SRMR for all three samples were <0.08, which indicates a good fit (21); and all of the RMSEA values for all three samples were 0.08 or less, which indicates a reasonably good fit (22). The values for the CFI, SRMR, and RMSEA were also similar to those obtained by Jones and Skaggs (13) using the 26-item version of the MUSIC Inventory (see Table 2). In fact, the values in the present study for the 20-item short-form were better than the 26-item version for the CFI and the RMSEA. In addition to the three different fit indices, we assessed the item standardized factor loadings from the CFA for each sample (see Table 4). Because the item loadings on each factor were much higher than 0.32 (ranging from 0.63 to 0.91), we concluded that the items loaded very well on the appropriate factors (24).

Based on values for the fit indices and factor loadings, we conclude that the data fit the five-factor structure of the MUSIC model well. This finding provides evidence for the construct validity of the MUSIC Inventory scales because it demonstrates that the items in each scale are distinct from the items in the other scales.

### Predictive Validity of the MUSIC Inventory

We assessed the predictive validity of the MUSIC Inventory by correlating the MUSIC Inventory scale scores with students' self-reported course effort and overall course rating. Researchers have documented that students' MUSIC perceptions in a course are related to their effort and course rating [e.g., (18)]; therefore, we expected the MUSIC scales to correlate positively with course effort and course rating.

#### Course Effort

Overall, the MUSIC scales were positively related to course effort, but the correlation coefficients were not very high (see Table 3). The effect size was small for almost all of the values and was insignificant for empowerment (all three samples) and caring (one of the samples); and, the success scale was negatively correlated with effort for two of the samples (samples 1 and 3). The correlations between the MUSIC scales and effort were higher in the Jones (18) study (4 correlations had a large effect size, 17 correlations had a medium effect size, five correlations had a small effect size, and two correlations were statistically insignificant) and in the Jones and Skaggs (13) study (three correlations had a medium effect size and two correlations had a small effect size).

To understand why effort was correlated with some of the MUSIC scales and not others, we examined the reasons students provided for the open-ended item (see Table 5). For the *usefulness* component of the MUSIC model, students put forth more effort when they wanted to learn the information because it was important for their future or education. Conversely, some students did not put forth effort when they found it useless to study. These qualitative findings are consistent with our quantitative correlational findings for which usefulness and effort were positively correlated in the three samples (*r* = 0.23, 0.24, and 0.44).

For the *interest* component of the MUSIC model, some students reported that they put forth effort because the course or material was interesting or enjoyable; or conversely, that they did not put forth effort when the topics were not interesting. These findings were consistent with the positive correlations between interest and effort in the three samples (*r* = 0.15, 0.26, and 0.45).

For the *success* component of the MUSIC model, it is impossible to determine students' success perceptions based on their responses to the open-ended item, with one exception (note that the success component of the MUSIC model refers to students' perceptions that they *can* succeed, not the extent to which they have actually succeeded). Some students reported that they did not put forth effort when they thought that the course was easy or when not much time was needed to study. Their belief that the course was easy implies that they believed that they could succeed. Therefore, they could have had high success perceptions, yet not had to put forth a lot of effort in the course, resulting in a negative correlation between the success and effort variables. Because the relationships between success perceptions and effort for other students in these classes could have been positive, negative, or insignificant, when all of the students were combined, it resulted in a small relationship between success perceptions and effort (*r* = −0.10, 0.12, and −0.20). Future studies could examine how students' perceptions of success interact with their perceptions of course difficulty to affect students' effort in courses.

The responses to the open-ended item did not include reasons that directly related to students' empowerment or caring. Consistent with this finding, the correlations between students' effort and empowerment were insignificant and the correlations were small for caring in two samples and insignificant in one sample. It is possible, however, that empowerment and caring affect students' effort indirectly through their relationships to the other MUSIC components.

In sum, veterinary medicine students are motivated by a variety of factors, some of which appear to be within the instructor's control (i.e., affecting students' MUSIC perceptions) and other factors that may not be, such as students' desire to achieve a high class ranking and to maintain a work-life balance. Being aware of these different motivations may help instructors to design instruction that not only engages students in the coursework, but also takes into consideration students' other motivations in life.

#### Course Rating

Across the three samples, two of the correlations between the MUSIC scales and course rating represented a large effect size, 10 represented a medium effect size, and three represented a small effect size (see Table 3). These correlations are similar in size to (or slightly smaller) than those presented in other studies (13, 18). These findings indicate that when students rate the MUSIC model components highly, they also rate the course highly, and vice versa. Although these correlations do not allow us to imply that the MUSIC perceptions *caused* the high ratings, it may be possible for instructors to intentionally select teaching strategies that increase students' MUSIC perceptions, which may lead to increases in students' perceptions of the course quality. In fact, researchers have conducted interventions intended to increase students' perceptions of one or more MUSIC model components and these interventions have resulted in increased motivation and engagement [for examples, see (34) and (35)].

## Implications and Conclusion

When used with veterinary medical students, the psychometric properties of the short-form College Student version of the MUSIC Inventory are acceptable. Assessing students' MUSIC perceptions with the MUSIC Inventory may be useful to instructors because they can use the results to develop teaching strategies that can affect students' MUSIC perceptions. At the class-level, *instructors* can use the scores from the MUSIC Inventory to enhance their instruction in areas that are rated lower. For example, if students' scores on the interest scale are low, instructors could choose one or more strategies to increase students' interest, such as varying instructional activities, involving students in discussions, or trying different teaching approaches [see (1) and (2), for more examples].

Instructors could also use the MUSIC Inventory scores at the individual-level, by examining individual students' scores to better understand why any one particular student is not putting forth effort in the course. For example, if a student reports low scores on the usefulness scale, an instructor could consider strategies to increase that student's perceptions of the usefulness of the content. At the program-level or college-level, the inventory could be used to compare students' perceptions across courses to identify strengths and weaknesses in particular courses, curricula, or teaching approaches.

Finally, *researchers* can use the inventory to assess students' perceptions of instruction for a variety of purposes. For example, researchers could survey students before and after an intervention. Researchers could also investigate the relationships between students' MUSIC perceptions and other antecedents and consequences, such as students' professional goals and identity [e.g., (36)].

## Ethics Statement

The Virginia Tech Institution Review Board (IRB) Chair, David M Moore, approved the New Application request for the research protocol. Students were provided with an Informed Consent Form (approved by the IRB) at the end of the online questionnaire that was used to collect the data. Only students who elected to participate in the study were included in the study. Because the study was determined by the IRB to involve only minimal risk, the protocol was approved as Exempt, under 45 CFR 46.110 category(ies) 1,2,4.

## Author Contributions

BJ and MB conceptualized the study and collected the data. BJ, MB, and MJ analyzed the data. BJ and MB drafted the manuscript with suggestions from MJ.

### Conflict of Interest Statement

The authors declare that the research was conducted in the absence of any commercial or financial relationships that could be construed as a potential conflict of interest.

## References

[1] Jones BD Motivating students to engage in learning: the MUSIC model of academic motivation. Int J Teach Learn High Edu. (2009) 21:272–85.

[2] Jones BD Motivating Students by Design: Practical Strategies for Professors, 2nd ed Charleston, SC: CreateSpace (2018).

[3] Jones BD Epler CM Mokri P Bryant LH Paretti MC The effects of a collaborative problem-based learning experience on students' motivation in engineering capstone courses. Interdisciplin J Problem-Based Learn. (2013) 7:34–71. 10.7771/1541-5015.1344

[4] Lee WC Kajfez RL Matusovich HM Motivating engineering students: evaluating an engineering student support center with the MUSIC model of academic motivation. J Women Minorities Sci Eng. (2013) 19:245–71. 10.1615/JWomenMinorScienEng.2013006747

[5] Dockter D Uvarov C Guzman-Alvarez A Molinaro M Improving preparation and persistence in undergraduate STEM: why an online summer preparatory chemistry course makes sense. In: Sorensen PM Canelas DA editors. Online Approaches to Chemical Education. Washington: DC, American Chemical Society (2017) p. 7–33.

[6] Mora CE Anorbe-Diaz B Gonzalez-Marrero AM Martin-Gutierrez J Jones BD Motivational factors to consider when introducing problem-based learning in engineering education courses. Int J Eng Edu. (2017) 33:1000–17.

[7] Hall S Jones BD Amelink C Hu D Educational innovation in the design of an online nuclear engineering curriculum. J Effect Teach. (2013) 13:58–72.

[8] Tu H-W Jones BD. Redesigning a neuroscience laboratory course for multiple sections: An action research project to engage students. J Undergrad Neurosci Edu. (2017) 15:A137–43. 28690435 PMC5480842

[9] McGinley J Jones BD A brief instructional intervention to increase students' motivation on the first day of class. Teach Psychol. (2014) 41:158–62. 10.1177/0098628314530350

[10] Jones BD Osborne JW Paretti MC Matusovich HM Relationships among students' perceptions of a first-year engineering design course and their engineering identification, motivational beliefs, course effort, and academic outcomes. Int J Eng Edu. (2014) 30:1340–56.

[11] Jones BD Tendhar C Paretti MC The effects of students' course perceptions on their domain identification, motivational beliefs, and goals. J Career Dev. (2016) 43:383–97.

[12] Jones BD User Guide for Assessing the Components of the MUSIC Model of Academic Motivation. (2017). Avaliable online at:http://www.theMUSICmodel.com

[13] Jones BD Skaggs GE Measuring students' motivation: validity evidence for the MUSIC model of academic motivation inventory. Int J Scholarsh Teach Learn. (2016) 10:1–9. 10.20429/ijsotl.2016.100107

[14] Tendhar C Singh K Jones BD Using the domain identification model to study major and career decision-making processes. Eur J Eng Edu. (2017) 43:235–46. 10.1080/03043797.2017.1329280

[15] Jones BD Li M Cruz JM A cross-cultural validation of the MUSIC^®^ model of academic motivation inventory: evidence from Chinese- and Spanish-speaking university students. Int J Edu Psychol. (2017) 6:366–85. 10.17583/ijep.2017.2357

[16] Mohamed HE Soliman MH Jones BD A cross-cultural validation of the MUSIC model of academic motivation and its associated inventory among Egyptian university students. J Counsel Q J. (2013) 36:2–14. 10.13140/RG.2.1.2532.9123

[17] Pace AC Ham A-JL Poole TM Wahaib KL Validation of the MUSIC^®^ model of academic motivation inventory for use with student pharmacists. Curr Pharm Teach Learn. (2016) 8:589–97. 10.1016/j.cptl.2016.06.001

[18] Jones BD An examination of motivation model components in face-to-face and online instruction. Electr J Res Edu Psychol. (2010) 8:915–44. 10.25115/ejrep.v8i22.1455

[19] Porter SR Whitcomb ME Weitzer WH Multiple surveys of students and survey fatigue. In: Porter SR editor. Overcoming Survey Research Problems. New Directions for Institutional Research, Vol. 121 San Francisco: Jossey-Bass (2004) p. 63–74.

[20] Kline RB Principles and Practice of Structural Equation Modeling (2005). New York, NY: Guilford Press.

[21] Hu LT Bentler PM Cutoff criteria for fit indices in covariance structure analysis: conventional criteria versus new alternatives. Struct Equ Model. (1999) 6:1–55. 10.1080/10705519909540118

[22] Browne MW Cudeck R Alternative ways of assessing model fit. In: Bollen KA Long JS editors. Testing Structural Equation Models. Newbury Park, CA: Sage (1993) p. 445.

[23] Byrne BM Structural Equation Modeling With AMOS: Basic Concepts, Applications, and Programming (2001). Mahwah, NJ: Lawrence Erlbaum.

[24] Tabachnick BG Fidell LS Using Multivariate Statistics, 3rd ed New York, NY: HarperCollins (1996).

[25] Cohen J Statistical Power Analysis for the Behavioral Sciences, 2nd ed Hillsdale, NJ: Erlbaum (1988).

[26] Glaser BG Strauss AL The Discovery of Grounded Theory: Strategies for Qualitative Research. Chicago, IL: Aldine (1967).

[27] Strauss A Corbin J Basics of Qualitative Research: Techniques and Procedures for Developing Grounded Theory. Thousand Oaks, CA: Sage (1998).

[28] Kline P Psychometrics and Psychology. London: Academic Press (1979).

[29] Hutcheson G Sofroniou N The Multivariate Social Scientist: Introductory Statistics Using Generalized Linear Models. Thousand Oaks, CA: Sage Publications (1999).

[30] Myers ND Ahn S Jin Y Same size and power estimates for a confirmatory factor analytic model in exercise and sport: a Monte Carlo approach. Res Q Exerc Sport (2011) 82:412–23. 10.1080/02701367.2011.10599773 21957699

[31] Nunnally JC Psychometric Theory, 2nd ed New York, NY: McGraw-Hill (1978).

[32] Gorsuch RL Factor Analysis, 2nd ed Hillsdale, NJ: Erlbaum (1983).

[33] Durik AM Shechter OG Noh M Rozek CS Harackiewicz JM What if I can't? Success expectancies moderate the effects of utility value information on situational interest and performance. Motiv Emot. (2015) 39:104–18. 10.1007/s11031-014-9419-0

[34] Brown ER Smith JL Thoman DB Allen JM Muragishi G. From bench to bedside: a communal utility value intervention to enhance students' biomedical science motivation. J Educ Psychol. (2015) 107:1116–35. 10.1037/edu0000033 26617417 PMC4657866

[35] Lazowski RA Hulleman CS Motivation interventions in education: a meta-analytic review. Rev Educ Res. (2016) 86:602–40. 10.3102/0034654315617832

[36] Jones BD Ruff C Osborne JW Fostering students' identification with mathematics and science. In: Renninger KA Nieswandt M Hidi S editors. Interest in Mathematics and Science Learning. Washington, DC: American Educational Research Association (2015). p. 331–52. 10.3102/978-0-935302-42-4_19

